# Regional cerebral oxygen saturation and postoperative delirium in endovascular surgery: a prospective cohort study

**DOI:** 10.1186/s13063-019-3586-y

**Published:** 2019-08-14

**Authors:** Xiaohua Wang, Kunpeng Feng, Haixia Liu, Yanhui Liu, Ming Ye, Guoguang Zhao, Tianlong Wang

**Affiliations:** 1 Department of Anesthesiology, Xuanwu Hospital, Capital Medical University, Beijing, 100053 China; 2 Institute of Geriatrics, Beijing, China; 3 National Clinical Research Center for Geriatric Disorders, Beijing, China; 4 Department of Cardiac surgery, Xuanwu Hospital, Capital Medical University, Beijing, 100053 China; 5 Department of Neurosurgery, Xuanwu Hospital, Capital Medical University, Beijing, 100053 China

**Keywords:** Delirium, Regional cerebral oxygen saturation, rSO_2_

## Abstract

**Background:**

Delirium is an acute mental disorder and common postoperative complication. Monitoring regional cerebral oxygen saturation (rSO_2_) in endovascular therapeutic surgery may allow real-time monitoring of cerebral desaturation, avoiding profound cerebral dysfunction, and reducing the incidence of delirium. We sought to examine the incidence of delirium in patients undergoing endovascular surgery.

**Methods:**

This was a clinical cohort trial (registered with http://www.clinicaltrials.gov [NCT02356133]). We monitored the rSO_2_ of 43 patients undergoing general anesthesia and cerebral endovascular surgery. The occurrence of delirium after surgery was recorded with the Confusion Assessment Method (CAM). Multivariate logistic regression was performed to identify the main predictor of delirium.

**Results:**

rSO_2_ was significantly different between the delirium and no-delirium groups. The occurrence of delirium was 35% in our cohort, and higher rSO_2_ desaturation scores were significantly associated with profound delirium (higher CAM score; odds ratio = 1.002; *P* = 0.021). The maximum declines of systolic blood pressure were 24.86 (21.78–27.93) and 32.98 (28.78–37.19) in the no-delirium and delirium groups, respectively, which were significantly different (*P* = 0.002) but not closely associated with delirium in multivariate analysis (*P* = 0.512). Anesthesia, mechanical ventilation duration, and having two vascular risk factors differed significantly between groups but were poorly associated with delirium outcome.

**Conclusions:**

Elevated rSO_2_ desaturation score was predictive of the occurrence of postoperative delirium following endovascular surgery. Monitoring rSO_2_ is invaluable for managing controlled hypotension during endovascular surgery and reducing postoperative delirium.

**Trial registration:**

ClinicalTrials.gov, NCT02356133. Registered 1 February 2015. All statistical analysis results submitted August 4, 2018.

**Electronic supplementary material:**

The online version of this article (10.1186/s13063-019-3586-y) contains supplementary material, which is available to authorized users.

## Background

Delirium is a complex neuropsychiatric syndrome that has a high prevalence in acute hospitals and is encountered across all healthcare settings. It is associated with adverse outcomes, including comorbidities and mortality. The incidence of postoperative delirium varies from 10% to 55%, and it is more common in patients with vascular dysfunction [1–3]. Previous studies indicated that several factors, such as age, education, ethanol consumption, preoperative cognitive status, and head trauma, are preoperative risk factors for delirium [4–9]. Intraoperative cerebral ischemia and cerebral oxygen desaturation have been proposed as possible mechanisms of postoperative cognitive dysfunction [10–12]. However, other important factors that have not been assessed, such as regional cerebral oxygen saturation (rSO_2_), hypotension, and hemoglobin, significantly affect cerebral perfusion and oxygen metabolism [13] and may further increase the risk of delirium. Delirium after neurosurgery is mainly caused by cerebral hypoperfusion and cerebral anoxia [14–17]. A study using rSO_2_ during carotid revascularization validated rSO_2_ as a potential reliable marker of hypoperfusion with a sensitivity of 100% and specificity of 90.6% [18]. Another study noted that near-infrared spectroscopy decline during the coiling of ruptured aneurysms was strongly associated with vasospasm during the procedure [19]. Monitoring rSO_2_ can quickly reflect changes in the balance between cerebral oxygen supply and demand. Early detection and management of predisposing risk factors for delirium would lead to improved outcomes for patients.

Endovascular therapy or interventions, also known as intra-arterial therapy, often require strict blood pressure control [20]. Cerebral aneurysm is a common cerebral vascular disease that requires endovascular surgery; long-term controlled hypotension is necessary during the perioperative period to ensure safe completion of the operation, especially in ruptured aneurysms [20]. Controlled hypotension during the surgical procedure reduces bleeding and improves visualization, but it may be related to postoperative cognitive dysfunction when cerebral perfusion is not maintained [21]. Intraoperative hypotension is an infrequent direct cause of cerebral oxygen desaturation and can contribute to injury caused by embolism or surgery, especially during the perioperative period. However, the appropriate blood pressure control regimen that leads to the most favorable clinical cerebral perfusion level in these patients remains inconclusive. Additionally, anesthesiologists have a significant challenge in maintaining the proper balance of rSO_2_. This requires real-time noninvasive monitoring that can directly reflect the relationship between cerebral oxygen supply and demand and cerebral perfusion.

In this prospective cohort study, our aim was to examine the perioperative risk factors associated with the development of delirium in patients following endovascular surgery. We sought to observe the appropriate range for rSO_2_ using noninvasive cerebral oxygen saturation monitoring and to assess control of hypotension during endovascular intervention surgeries to reduce the incidence of postoperative delirium. Our hypothesis was that rSO_2_ would correlate with postoperative delirium after cerebral endovascular surgery.

## Methods

### Patient characteristics and power calculations

**Fig. 1:**
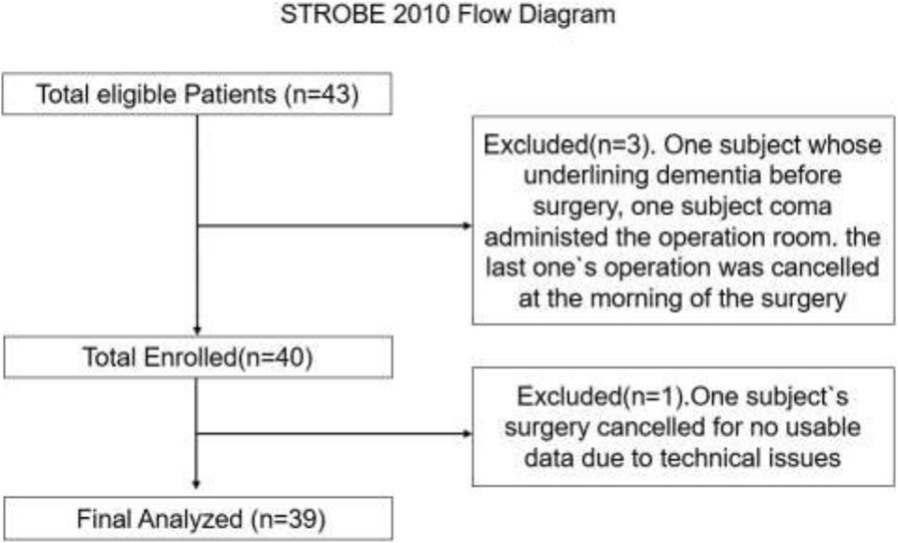
Flow diagram of the patient selection process

This prospective observational study was approved by our university’s institutional review board (IRB 0810-758). Written informed consent was obtained from all patients participating in the trial. The trial was registered with http://www.clinicalTrials.gov (NCT02356133; date of registration, February 1, 2015). Our methodology followed the international guidelines for observational studies. The trial was conducted and reported according to the Strengthening the Reporting of Observational Studies in Epidemiology (STROBE) 2010 statement (Fig. 1 and Additional file 1: Table S1). We used G*Power to conduct power analysis. To calculate the required study size, we considered the results of previous studies performed in a similar population. To detect time points in mean rSO_2_ values recorded during surgery, accepting a two-tailed α error of 5% and β error of 10%, 39 patients were required. Generally, the limiting values for the reference interval are the 0.025 and 0.975 fractiles of the results distribution in the population. In the present study, only high rSO_2_ desaturation score was likely to be of clinical interest. Therefore, the use of the 0.05 fractile as the high reference limit made the most sense [22, 23]. We recruited 43 prospective consecutive patients with intracranial aneurysms who were scheduled for endovascular surgery with general anesthesia between May 1, 2015, and January 1, 2017, at Xuanwu Hospital. Inclusion criteria were as follows: (1) elective intracranial aneurysm embolization, (2) 35–70 years old, (3) American Society of Anesthesiologists (ASA) physical status score of II–IV, and (4) body mass index (BMI) ranging from 22 to 45 kg·m^− 2^. Exclusion criteria were as follows: (1) preexisting neuropsychiatric disorders or inability to correctly perform neurocognitive tests on the patient, (2) emergency operation, (3) diagnosis of coma, (4) depression, or (5) cognitive impairment.

### Anesthetic technique and protocol

Patients’ demographic characteristics were recorded. After admission to the operating room (OR), patients were administered 8 ml·kg^− 1^ Ringer solution with an intraoperative maintenance dose of 4 ml·kg·h^− 2^. Patients were monitored with a five-lead electrocardiogram (ECG), invasive arterial pressure measurements, oxygen saturation measured by pulse oximetry (SpO_2_), and rSO_2_. Baseline blood pressure, heart rate, and rSO_2_ were acquired 15 min after admission to the OR. Anesthetic induction was performed using intravenous etomidate (0.2 mg·kg^− 1^), sufentanil (0.3 μg·kg^− 1^), and rocuronium (0.5 mg·kg^− 1^). Tracheal intubation was performed for mechanical ventilation when satisfactory muscle relaxation was achieved (approximately 3 min), followed by connection to an anesthesia machine (Datex*-*Ohmeda Avance S5; GE Healthcare, Chicago, IL, USA) for volume control ventilation with a tidal volume of 8 ml·kg^− 1^, respiratory rate of 12·min^− 1^, inspiration/expiration ratio of 1:2, and end-tidal carbon dioxide pressure (PETCO_2_) of 35–40 mmHg. SpO_2_ was maintained at 99–100% during the general anesthesia period. An infusion of 0.06 mg·kg·min^− 2^ rocuronium, 0.3 mg·kg·min^− 2^ propofol, 0.3 μg·kg·min^− 2^ remifentanil, and 0.4–0.6% sevoflurane was administered continuously during surgery. Nimodipine (1–3 ml·h^− 1^) was administered to control hypotension during the operation.

### Perioperative monitoring and data acquisition

The following variables were recorded for all patients: general patient data, including age, sex, weight, height, BMI, preoperative hemoglobin, and ASA physical status; and medical history of hypertension, diabetes mellitus, coronary artery disease, and stroke. Patients who had two systemic vascular comorbidities (including history of hypertension, diabetes mellitus, coronary disease, and/or stroke) were classed as having two risk factors; those with three systemic vascular comorbidities (including history of hypertension, diabetes mellitus, coronary disease, and/or stroke) were classed as having three risk factors. During the operation, we monitored standard noninvasive vital signs, including systolic blood pressure (SBP), diastolic blood pressure (DBP), ECG data, SpO_2_, and PETCO_2_. In addition, we recorded anesthesia, mechanical ventilation, and operation duration. Finally, rSO_2_ indices were continuously recorded in the brain during the operation using a cerebral oximeter (C2030C; CAS Medical Systems Inc., Branford, CT, USA). Briefly, bilateral rSO_2_ probes were placed on the patient’s forehead and stabilized. Cerebral oxygen data were recorded bilaterally every minute. The maximum SBP (%) and DBP (%) declines were calculated as follows: ([lowest SBP recorded during the operation – SBP baseline score]/SBP baseline score) × 100. ([lowest DBP recorded during the operation – DBP baseline score]/DBP baseline score) × 100. Baseline SBP and DBP both indicate preoperative levels 15 min after entry into the OR.

### rSO_2_ desaturation score

To relate intraoperative rSO_2_ to clinical outcomes and hemodynamic variables, we used the rSO_2_ desaturation score as a measure of the degree of desaturation, where the score was calculated using the baseline and recorded rSO_2_ over time [24–26]. The rSO_2_ desaturation score is expressed as a product with the units of percentage per minute, as described below. M = measured rSO_2_; B = baseline rSO_2_. Z is calculated as the measured rSO_2_ – threshold rSO_2_ according to the following definition: if M < B, then Z = M; if M ≥ B, then Z = 0. The rSO_2_ desaturation score for each patient was calculated using the following formula: rSO_2_ desaturation score = (∑ Z) × t, where t = total number of minutes from anesthesia induction until exit from the OR [24–26], which accounts for both depth and duration of desaturation below the baseline of each patient. Complete rSO_2_ datasets were acquired for 39 patients. For patients with bilateral cerebral rSO_2_ sensors, mean rSO_2_ was used for analysis.

### Postoperative delirium assessment

We used the Confusion Assessment Method (CAM) delirium score to evaluate the degree of delirium [27]. Diagnosis of delirium was assessed using an algorithm based on the CAM [28]. CAM diagnostic criteria included attention dysfunction, confusion, level of consciousness, disorientation, memory loss, perception dysfunction, psychomotor excitement and retardation, volatility, and sleep-wake cycle changes [29]. Rapid CAM diagnosis of delirium requires the following four key delirium characteristics: (1) acute onset and disease fluctuations, (2) inattention, (3) disordered thinking, and (4) changes in the level of consciousness (any state of consciousness other than fully conscious). The diagnosis of delirium requires the presence of (1) and (2), accompanied by (3) or (4) or both [24]. The diagnosis of delirium was performed by three nurses who had undergone training for 1 week to ensure consistency.

### Statistical analysis

IBM SPSS Statistics software (version 22.0; IBM, Armonk, NY, USA) was used to analyze the data. The patients were divided into two groups according to the incidence of delirium. The presence or absence of delirium was evaluated as a dichotomous variable. Continuous variables were expressed as mean and 95% confidence interval (95% CI) and were compared using *t* tests. Categorical data were expressed as frequency and percentage and were compared using chi-square tests. The distribution of data was evaluated using the Kolmogorov-Smirnov test. Significant variables from the *t* and chi-square tests were included in Spearman’s rank correlation analysis. Logistic regression analysis was performed to analyze the association between delirium and predictors correlated with delirium.

## Results

### Patient demographics and perioperative variability between groups

On the basis of the inclusion and exclusion criteria, 39 patients were analyzed in our study. Four patients were excluded for the following reasons: an underlying dementia diagnosis (*n* = 1), coma (*n* = 1), and canceled operation (*n* = 1). Figure 1 shows the selection procedure for the patients. A total of 38 patients were safely discharged from the hospital after surgery; one patient experienced severe delirium and died on the fifth day after surgery. Postoperative delirium occurred in 14 of 39 patients (35%). There were no different of age, height, weight, BMI, Hemoglobin, and ASA physical status in delirium group and no delirium group (Table 1). Preoperatively, there were no significant differences between the groups in terms of patient sex or history of hypertension, diabetes mellitus, coronary disease, and stroke. The number of patients with two risk factors was significantly lower in the no-delirium group (*P* = 0.010) (Table 2). There was no significant difference in operation duration (*P* = 0.147) or maximum DBP decline (*P* = 0.300) between groups (Table 3). Mechanical ventilation duration and anesthesia duration were significantly higher in the delirium group (mechanical ventilation duration, 143.85; 100.51–187.18; anesthesia duration, 168.46; 127.06–209.86) than in the no-delirium group (mechanical ventilation duration, 118.65; 105.18–132.13, *P* = 0.044; anesthesia duration, 133.46; 120.13–146.79; *P* = 0.023) (Table 3). The maximum SBP decline was significantly higher in the delirium group (32.98; 28.78–37.19) than in the no-delirium group (24.86; 21.78–27.93; *P* = 0.002) (Table 3). Furthermore, rSO_2_ desaturation score was significantly higher in the delirium group (1267.44; 866.82–1668.07) than in the no-delirium group (231.95; 55.70–408.21; *P* = 0.001) (Table 3).

**Table 1:** Demographic and preoperative characteristics of the delirium and no delirium groups

Variable	Delirium (no) (*n* = 25)	Delirium (yes) (*n* = 14)	*P* value
Age (years)	52.62 (47.72–57.51)	54.08 (46.16–62.00)	0.731
Height (cm)	168.19 (165.42–170.96)	164.54 (160.78–168.29)	0.114
Weight (kg)	73.62 (68.21–79.02)	70.31 (66.89–73.73)	0.401
BMI (kg∙m^− 2^)	25.85 (24.48–27.22)	25.99 (24.63–27.34)	0.892
Hemoglobin (g/L)	142.04 (136.13–147.95)	140.23 (133.75–146.71)	0.300
ASA physical status	2.983 (2.121–3.165)	2.891 (2.753–3.521)	0.580

*BMI* Body mass index, ASA American Society of Anesthesiologists

Data are expressed as mean (95% confidence interval), based on *t* test and chi-square test for analysis

**Table 2:** Patient characteristics and preoperative clinical prediction of delirium

Variable	Delirium (no) (*n* = 25)	Delirium (yes) (*n* = 14)	*P* value
Male sex, *n* (%)	18 (46.15%)	7 (17.95%)	0.104
History of hypertension	8 (20.51%)	6 (15.38%)	0.816
History of diabetes mellitus	1 (2.56%)	4 (10.26%)	0.261
History of coronary artery disease	0 (0)	3 (7.69%)	0.255
History of stroke	1 (2.56%)	3 (7.69%)	0.065
Two risk factors	1 (2.56%)	7 (17.95%)	0.010*
Three risk factors	1 (2.56%)	2 (5.13%)	0.254

Data are expressed as number (%), analyzed using chi-square test

*P < 0.05

**Table 3:** Intraoperative clinical factors of the delirium and no-delirium groups

Variable	Delirium (no) (*n* = 25)	Delirium (yes) (*n* = 14)	*P* value
Operation duration (min)	101.35 (87.37–115.32)	123.08 (81.69–164.46)	0.147
Anesthesia duration (min)	133.46 (120.13–146.79)	168.46 (127.06–209.86)	0.023*
Mechanical ventilation duration (min)	118.65 (105.18–132.13)	143.85 (100.51–187.18)	0.044*
Maximum decline in SBP (%)	24.86 (21.78–27.93)	32.98 (28.78–37.19)	0.002**
Maximum decline in DBP (%)	23.75 (20.01–27.49)	25.71 (18.69–32.74)	0.300
rSO_2_ desaturation score	231.95 (55.70–408.21)	1267.44 (866.82–1668.07)	0.001**

*rSO*_*2*_ Regional cerebral oxygen saturation, *SBP* Systolic blood pressure, *DBP* Diastolic blood pressure

Data expressed as mean (95% confidence interval), based on *t* test

*P < 0.05

**P < 0.01

### Correlations between delirium and different variables

The difference in rSO_2_ desaturation score between the groups was closely associated with delirium (*r* = 0.597; *P* = 0.001) (Table 4). Furthermore, maximum SBP decline was significantly correlated with delirium (*r* = 0.465; *P* = 0.003) (Table 4). The incidence of two systemic vascular risk factors was also correlated with delirium (*r* = 0.486; *P* = 0.002) (Table 4). However, anesthesia duration (in minutes) and mechanical ventilation duration (in minutes) were not correlated with delirium (anesthesia duration, *r* = 0.183; *P* = 0.264; mechanical ventilation duration, *r* = 0.114; *P* = 0.489) (Table 4).

**Table 4:** Spearman correlation analysis of the different predictors of delirium

Variable	*r*	Significance
rSO_2_ desaturation score	0.597	0.001**
Maximum decline in SBP (%)	0.465	0.003**
Anesthesia duration (min)	0.183	0.264
Mechanical ventilation duration (min)	0.114	0.489
Two risk factors	0.486	0.002**

*rSO*_*2*_ Regional cerebral oxygen saturation, *SBP* Systolic blood pressure

**P < 0.01

### Logistic regression analysis of the preoperative predictors of delirium

Logistic regression analysis revealed that rSO_2_ desaturation score was a significant positive predictor of postoperative delirium (OR = 1.002; *P* = 0.021) (Table 5), suggesting that an increase in rSO_2_ desaturation was a surrogate for increased cerebral ischemia and insufficient oxygen supply, leading to an increased risk of postoperative delirium. Table 5 summarizes the results of logistic regression analysis between the potential predictors for delirium. There was no significant association between maximum SBP decline and delirium based on multivariate logistic regression (OR = 1.059; *P* = 0.512). There was a very weak association between the incidence of two systemic vascular risk factors and postoperative delirium; however, this did not reach statistical significance (OR = 3.593; *P* = 0.361) (Table 5). To determine the appropriate reference intervals for rSO_2_ scores for the best monitoring of controlled hypoperfusion, we calculated the 95% prediction interval based on the one-sided high reference limit among healthy individuals. We found that the threshold rSO_2_ desaturation score was 493.96, indicating that a person with a value above this reference limit had a higher risk of delirium.

**Table 5:** Multivariate logistic regression analysis of the predictors of delirium

Variable	β	SE	Wald estimate	*df*	*P* value	Exp(B) (OR)	95% CI	
							Lower	Upper
rSO_2_ desaturation score	0.002	0.001	5.290	1	0.021*	1.002	1.000	1.004
Maximum decline in SBP	0.058	0.088	0.429	1	0.512	1.059	0.892	1.258
Two risk factors	1.279	1.400	0.834	1	0.361	3.593	0.231	55.915

*rSO*_*2*_ Regional cerebral oxygen saturation, *SBP* Systolic blood pressure, *CI* Confidence interval, *OR* Odds ratio

*P < 0.05

## Discussion

Our results provide support for rSO_2_ desaturation score as an independent predictor of postoperative delirium following high-risk cerebral endovascular interventions of intracranial aneurysm. Measuring rSO_2_ is a noninvasive technique that can be used for bedside monitoring of cerebral oxygen saturation. It offers real-time data acquisition, excellent sensitivity, rapidity, and persistence. It is currently the most widely used method for monitoring cerebral oxygen saturation [29]. In recent years, there has been a significant increase in the use of rSO_2_ perioperatively, especially in cardiac, aortic, carotid, thoracic, neonatal, and geriatric surgeries, as well as in resuscitation [24, 30, 31]. The increasing use of rSO_2_ in clinical practice has allowed the maintenance of a specific rSO_2_ range shortening the length of tracheal extubation and duration of intensive care unit and hospital stay [32–34]. Appropriate maintenance of rSO_2_ contributes to improved patient outcomes, reduces the occurrence of postoperative neurological dysfunction, and decreases mortality rates [35, 36]. Our findings suggest that cerebral oximetry with near-infrared spectroscopy allows the estimation of brain tissue oxygenation that can directly predict delirium. In our research, the rSO_2_ desaturation score over time was used as a key index to directly evaluate cerebral oxygen supply status of patients and served as a predictor of postoperative delirium.

Previous research has reported that delirium results in prolonged hospital stay, increased medical costs, increased postoperative complications, increased mortality, and decreased functional status after discharge [37–40]. Mortality is increased by 11% for every additional 48 h of active delirium, highlighting the requirement for timely detection and treatment. In our study, the incidence of early postoperative delirium was 35%, which is consistent with the findings of previous studies [41, 42] and lower than the incidence of delirium among patients with cancer (49.8%) or trauma ICU patients (38.7%) [15, 43]. We described the incidence of delirium using an algorithm based on the CAM. This tool was preferred because it has been validated previously and is the gold standard for the diagnosis of delirium [44, 45].

In addition, we observed that the degree of maximum SBP decline was higher in the delirium group than in the nondelirium group, which indirectly suggested that a hypotensive and hypoperfusion state may affect the postoperative incidence of delirium. Hypotension is the main physiological mechanism for reducing cerebral perfusion and oxygenation. Controlled hypotension is widely used during endovascular surgical procedures to reduce bleeding and improve the procedure of micronavigation and embolization [46, 47]. There are three reasons underscoring the importance of controlling hypotension for aneurysm endovascular surgery. First, high blood pressure increases the incidence of bleeding, surgery failure rates, and risk of aneurysm rupture [48]. Second, cerebral hyperperfusion syndrome may be caused by rapidly increased blood flow into chronically hypoperfused parenchyma with resultant impaired autoregulation, which has been noted following the clipping of intracranial aneurysms and carotid stenting. Therefore, hypotension is an intraprocedural technique that can decrease the risk of cerebral hyperperfusion syndrome [49]. Third, clinically controlled hypotension prevents wire-induced vessel injury during endovascular therapy because blood pressure fluctuates significantly in patients with fragile vascular status [50].

However, blood pressure that is too low affects cerebral perfusion and oxygenation, which carries an increased risk in patients with cerebral vascular dysfunction. Previous studies have found that reduction in SBP to 140 mmHg can lead to delirium and poorer outcomes in endovascular surgery patients [51]. Judging the appropriate nadirs of blood pressure is crucial for avoiding excessive hypotension. One study reported that hypotension occurred in 99% of patients undergoing surgery, but only 10% of patients experienced cerebral desaturation (defined as a 20% reduction in rSO_2_ from baseline). Hypotension was observed almost 100 times as often as cerebral desaturation [11]. This indicates that hypotension is not fully coincidental with cerebral desaturation, of which the direct outcome is delirium. Our results revealed a drop in maximum SBP, with a difference of approximately 10 mmHg between groups. Nevertheless, no contribution of delirium was observed in multivariate logistic regression analysis of the predictors of delirium. Therefore, it is important to consider the optimal range for controlled hypotension to ensure appropriate brain perfusion. The current lack of certain safety limits for controlling hypotension suggests that direct monitoring to measure end organ perfusion or a validated surrogate measure may be more useful in endovascular surgery. Our data indicated that early and continuous rSO_2_ monitoring is a particularly important strategy to detect and further protect patients from delirium.

The etiology of delirium can be multifactorial. We observed that anesthesia and mechanical ventilation durations were significantly different between groups; therefore, anesthesia accumulation may be a significant risk for delirium, as reported in other studies [52]. These factors may indirectly affect delirium outcome. The potential mechanisms underlying their impact on delirium may include hemodynamic changes, neurotoxicity, delays in treatment, or prolonged intubation. Medical history of hypertension, diabetes mellitus, coronary artery disease, and stroke indicates a fragile vascular status. Our research demonstrated that a combination of two of these factors increased the risk of postoperative delirium. Patients with systemic vascular disease have a reduced tolerance for a decline in cerebral blood flow during anesthesia. Furthermore, there is an increased risk of cerebral hypoperfusion in systemic vascular disease, which may lead to cerebral metabolic dysfunction.

We determined rSO_2_ using near-infrared spectroscopy, which provided an objective measure of the supplementation and consumption of oxygen in the brain. In summary, we observed that changes in the rSO_2_ desaturation score were closely associated with the occurrence of delirium following cerebral endovascular intervention. We argue that treatments to prevent cerebral oxygen saturation falling below the rSO_2_ desaturation score threshold of 493.96 should be considered to prevent postoperative delirium in patients undergoing endovascular surgery. rSO_2_ is an important parameter for minimizing delirium and subsequent negative adverse events in high-risk cerebral vascular interventions. Our findings support intraoperative monitoring of cerebral oxygen saturation as a guide for controlled hypotension, which will reduce the risk of postoperative delirium.

There are two limitations of this study. All patients were recruited from a single center. Second, we observed that anesthesia and mechanical ventilation durations were significantly different between groups. We are planning further studies to address this and plan to record total anesthesia load to enable a more detailed assessment of the effects of anesthesia.

## Conclusions

In conclusion, this study demonstrated that rSO_2_ was a useful monitoring tool for anesthesiologists to maintain and achieve ideal hypotension and ensure cerebral perfusion and oxygenation in patients undergoing cerebral endovascular intervention.

## Additional file

Additional file 1: STROBE statement: checklist of items that should be included in reports of cohort studies. (DOC 84 kb)

## Data Availability

The raw data of this study are available from the corresponding author on reasonable request.

## Abbreviations

ASA: American Society of Anesthesiologists
BMI: Body mass index
CAM: Confusion Assessment Method
CI: Confidence interval
DBP: Diastolic blood pressure
ECG: Electrocardiogram
OR: Odds ratio
OR: Operating room
PETCO_2_: End-tidal carbon dioxide pressure
rSO_2_: Regional cerebral oxygen saturation
SBP: Systolic blood pressure
SpO_2_: Oxygen saturation measured by pulse oximetry
