# Supplementary material for: Regional cerebral oxygen saturation and postoperative delirium in endovascular surgery: a prospective cohort study
Source: Trials. 2019 Aug 14;20:504. doi: 10.1186/s13063-019-3586-y (PMC6694555; doi:10.1186/s13063-019-3586-y)
Supplement: Supplementary file 1 — STROBE statement: checklist of items that should be included in reports of cohort studies. (DOC 84 kb) [file 13063_2019_3586_MOESM1_ESM.doc]

STROBE Statement—Checklist of items that should be included in reports of ***cohort studies***

|  | Item No | Recommendation |
| --- | --- | --- |
| **Title and abstract** | 1 | (*a*) Title:Regional cerebral oxygen saturation and post-operation delirium in endovascular surgery: a prospective cohort study *(Indicate the study’s design with a commonly used term in the title or the abstract)* |
|  |  | (*b*)abstract: Background:Delirium is an acute mental disorder and common postoperative complication. Monitoring regional cerebral oxygen saturation (rSO2) in endovascular therapeutic surgery may allow real-time monitoring of cerebral desaturation, and in turn, avoid profound cerebral dysfunction and reduce incidences of delirium. We sought to examine the incidence of delirium in patients undergoing endovascular surgery.Methods: This was a clinical cohort trial [registered at clinicaltrial.gov (NCT-02356133)]. We monitored the rSO2 of 43 patients undergoing general anesthesia and cerebral endovascular surgery. The occurrence of delirium after surgery was recorded with the confusion assessment method (CAM). A multivariate logistic regression was performed to identify the main predictor of delirium.Results:rSO2 was significantly different between the delirium and no delirium groups. The occurrence of delirium was 35% in our cohort, and higher rSO2 Desaturation Scores were significantly associated with a profound outcome of delirium (higher CAM score; Odds Ratio = 1.002; P = 0.021). The maximum decline of systolic blood pressure (SBP) was 24.86 (21.78–27.93) and 32.98 (28.78–37.19) in the no delirium and delirium groups, respectively, which was significantly different (P = 0.002); however, was not closely associated with delirium on multivariate analysis (P = 0.512). Anesthesia, mechanical ventilation duration, and having 2 system vascular risk factors were different between the two groups; however, they were poorly associated with delirium outcome. *(Provide in the abstract an informative and balanced summary of what was done and what was found)* |
| Introduction | | |
| Background/rationale | 2 | Background:Delirium is an acute mental disorder and common postoperative complication. Monitoring regional cerebral oxygen saturation (rSO2) in endovascular therapeutic surgery may allow real-time monitoring of cerebral desaturation, and in turn, avoid profound cerebral dysfunction and reduce incidences of delirium. We sought to examine the incidence of delirium in patients undergoing endovascular surgery.*(Explain the scientific background and rationale for the investigation being reported)* |
| Objectives | 3 | In this prospective cohort study, we sought to examine the perioperative risk factors associated with the development of delirium in patients following endovascular surgery. We sought to observe the appropriate range for rSO2 using noninvasive cerebral oxygen saturation monitoring and assessing for regional hypotension during endovascular intervention surgeries to reduce the incidence of postoperative delirium. Our hypothesis was the rSO2 may correlate with postoperative delirium after cerebral endovascular surgery.*(State specific objectives, including any prespecified hypotheses)* |
| Methods | | |
| Study design | 4 | *Yes, we present key elements of study design early in the paper.*(present key elements of study design early in the paper) |
| Setting | 5 | We recruited 43 prospective consecutive patients with intracranial aneurysm who were scheduled for endovascular surgery with general anesthesia from 1 May 2015 to 1 January 2017 at the Xuanwu Hospital. *(Describe the setting, locations, and relevant dates, including periods of recruitment, exposure, follow-up, and data collection)* |
| Participants | 6 | (*a*) Inclusion criteria were as follows: elective intracranial aneurysm embolization; age, 35–70 years; American Society of Anesthesiologists (ASA) Physical Status score of II–IV; and body mass index (BMI) ranging from 22–45 kg·m2.*(Give the eligibility criteria, and the sources and methods of selection of participants. Describe methods of follow-up)* |
|  |  | (*b*) This was not matched studies. *(For matched studies, give matching criteria and number of exposed and unexposed)* |
| Variables | 7 | The first outcome was delirum occurence, There was no second outcomes in our research. The exposure was rSO2 Desaturation and SBP, DBP decline, and 2/3 systemic vascular risk factor. The potential confounders was gender, but no different in our research groups. The diagnostic criteria was show in CAM table. *(Clearly define all outcomes, exposures, predictors, potential confounders, and effect modifiers. Give diagnostic criteria, if applicable)* |
| Data sources/ measurement | 8* | rSO2 desaturation score was show clearly in method part. Diagnosis of delirium was assessed using an algorithm based on the Confusion Assessment Method (CAM) and was show in supplymentary table*. (For each variable of interest, give sources of data and details of methods of assessment (measurement). Describe comparability of assessment methods if there is more than one group)* |
| Bias | 9 | The diagnosis of delirium was performed by three nurses who had undergone training for one week to enable consistency. *(Describe any efforts to address potential sources of bias)* |
| Study size | 10 | We utilized G*Power for conducting a power analysis. To calculate the required study size, we considered the results of previous studies performed in a similar population. To detect time points in mean rSO2 values recorded during surgery accepting a two-tailed α error of 5% and a β error of 10%, 39 patients were required. Generally, the limiting values for the reference interval are the 0.025 and 0.975 fractiles of the result distribution in the population. In current study, only high rSO2 Desaturation Score are likely to be of clinical interest, and therefore, the use of the 0.05 fractile as high reference limit makes the most sense.(*Explain how the study size was arrived at)* |
| Quantitative variables | 11 | Continuous variables were expressed as mean and 95% confidence interval (95% CI) and were compared using t-tests.The logistic regression analysis was performed to analyze the association between the delirium and predictors that were correlated with delirium. *(Explain how quantitative variables were handled in the analyses. If applicable, describe which groupings were chosen and why)* |
| Statistical methods | 12 | (*a*)The patients were divided into two groups, according to the incidence of delirium. The presence or absence of delirium was evaluated as a dichotomous variable. Continuous variables were expressed as mean and 95% confidence interval (95% CI) and were compared using t-tests. Categorical data were expressed as frequency and percentage and were compared using χ2 tests.*(Describe all statistical methods, including those used to control for confounding)* |
|  |  | (*b*)The distribution of data was evaluated using the Kolmogorov-Smirnov test. The significant variables from the t- and χ2 tests were included in Spearman’s rank correlation analysis. The logistic regression analysis was performed to analyze the association between the delirium and predictors that were correlated with delirium. (*Describe any methods used to examine subgroups and interactions)* |
|  |  | (*c*) The missing data in our research lower than 98%. The missing data was systemly ignore in the data analysis in SPSS software  *(Explain how missing data were addressed)* |
|  |  | (*d*) We show it in the figure -1 *(If applicable, explain how loss to follow-up was addressed)* |
|  |  | (*e*) There was no sensitivity analyses in our research. *(Describe any sensitivity analyses)* |
| Results | | |
| Participants | 13* | (a) We already show this in figure-1. *(Report numbers of individuals at each stage of study—eg numbers potentially eligible, examined for eligibility, confirmed eligible, included in the study, completing follow-up, and analysed)* |
|  |  | (b)We already show this in figure-1. *(Give reasons for non-participation at each stage)* |
|  |  | (c) We already show this in figure-1. *(Consider use of a flow diagram)* |
| Descriptive data | 14* | (a) We recruited 43 prospective consecutive patients with intracranial aneurysm who were scheduled for endovascular surgery with general anesthesia from 1 May 2015 to 1 January 2017 at the Xuanwu Hospital. Inclusion criteria were as follows: elective intracranial aneurysm embolization; age, 35–70 years; American Society of Anesthesiologists (ASA) Physical Status score of II–IV; and body mass index (BMI) ranging from 22–45 kg·m2. *(Give characteristics of study participants (eg demographic, clinical, social) and information on exposures and potential confounders)* |
|  |  | (b) There was only one participants contain missing data in SBP data in our research.Total missing data lower than 98% totally. *(Indicate number of participants with missing data for each variable of interest)* |
|  |  | (c) As we already show in method part, the follow-up time was 24 hours. *(Summarise follow-up time (eg, average and total amount))* |
| Outcome data | 15* | As we describe in the article, The first outcome was delirium (24hours), the second outcome was sever combination include death (10 days). *(Report numbers of outcome events or summary measures over time)* |
| Main results | 16 | (*a*) We reflect the all datas and analyezed results using 95% confidence interval *(Give unadjusted estimates and, if applicable, confounder-adjusted estimates and their precision (eg, 95% confidence interval). Make clear which confounders were adjusted for and why they were included)* |
|  |  | (*b*) We already report the category boundaries of rSO2 when this continuous variable were categorized by desaturation or no desaturation. *(Report category boundaries when continuous variables were categorized)* |
|  |  | (*c*) We note refer relative risk in our result. *(If relevant, consider translating estimates of relative risk into absolute risk for a meaningful time period)* |
| Other analyses | 17 | The significant variables from the t- and χ2 tests were included in Spearman’s rank correlation analysis. The logistic regression analysis was performed to analyze the association between the delirium and predictors that were correlated with delirium. *(Report other analyses done—eg analyses of subgroups and interactions, and sensitivity analyses)* |
| Discussion | | |
| Key results | 18 | We already summarise the key results in the discussion part. *(Summarise key results with reference to study objectives)* |
| Limitations | 19 | We already summarise the limiation in the discussion part. *(Discuss limitations of the study, taking into account sources of potential bias or imprecision. Discuss both direction and magnitude of any potential bias)* |
| Interpretation | 20 | We already discuss the detail in discussion part. *(Give a cautious overall interpretation of results considering objectives, limitations, multiplicity of analyses, results from similar studies, and other relevant evidence)* |
| Generalisability | 21 | We already discuss the generalisability in last paragraph of discussion part. *(Discuss the generalisability (external validity) of the study results)* |
| Other information | | |
| Funding | 22 | The funding of this article was show in the last paragraph. *(Give the source of funding and the role of the funders for the present study and, if applicable, for the original study on which the present article is based)* |

*Give information separately for exposed and unexposed groups.

**Note:** An Explanation and Elaboration article discusses each checklist item and gives methodological background and published examples of transparent reporting. The STROBE checklist is best used in conjunction with this article (freely available on the Web sites of PLoS Medicine at http://www.plosmedicine.org/, Annals of Internal Medicine at http://www.annals.org/, and Epidemiology at http://www.epidem.com/). Information on the STROBE Initiative is available at http://www.strobe-statement.org.
